# Reduced iNKT cells numbers in type 1 diabetes patients and their first‐degree relatives

**DOI:** 10.1002/iid3.79

**Published:** 2015-08-18

**Authors:** Nonantzin Beristain‐Covarrubias, Elsy Canche‐Pool, Rita Gomez‐Diaz, Luvia E. Sanchez‐Torres, Vianney Ortiz‐Navarrete

**Affiliations:** ^1^ Department of Molecular BiomedicineCenter for Research and Advanced Studies (CINVESTAV)Mexico CityMexico; ^2^ Immunology LaboratoryCenter for Regional Investigations “Dr. Hideyo Noguchi”MéridaMexico; ^3^ Research Unit on Clinical Epidemiology (UMAE), Specialty Hospital, National Medical CenterMexican Social Security InstituteMexico CityMexico; ^4^ Department of Immunology, National School of Biological ScienceNational Polytechnic InstituteMexico CityMexico

**Keywords:** Activation markers, autoimmune diabetes, CD355, CRTAM, invariant natural killer T cells

## Abstract

Type 1 diabetes (T1D) is an autoimmune disease that is characterized by the specific destruction of insulin‐producing pancreatic β cells. Invariant natural killer T (iNKT) cells have been associated with development of T1D. Class I MHC‐restricted T cell‐associated molecule (CRTAM) is expressed on activated iNKT, CD8^+^, and CD4^+^ T cells, and it is associated with the pro‐inflammatory profiles of these cells. *Crtam* gene expression in CD3^+^ lymphocytes from non‐obese diabetic (NOD) mice is associated with T1D onset. However, expression of CRTAM on T cells from patients with T1D has not yet been evaluated. We compared iNKT cell (CD3^+^Vα24^+^Vβ11^+^) numbers and CRTAM expression in a Mexican population with recent‐onset T1D and their first‐degree relatives with control families. Remarkably, we found lower iNKT cell numbers in T1D families, and we identified two iNKT cell populations in some of the families. One iNKT cell population expressed high iTCR levels (iNKT^hi^), whereas another expressed low levels (iNKT^lo^) and also expressed CRTAM. These findings support a probable genetic determinant of iNKT cell numbers and a possible role for these cells in T1D development. This study also suggests that CRTAM identifies recently activated iNKT lymphocytes.

## Introduction

Type 1 diabetes (T1D) is a chronic autoimmune disease that is characterized by the specific destruction of insulin‐producing pancreatic β cells, resulting in the loss of glycemic control. This destruction is mediated by auto‐reactive CD8^+^ and CD4^+^ T cells that produce a pro‐inflammatory response 1.

Invariant natural killer T (iNKT) cells are T lymphocytes that co‐express an invariant T cell receptor (iTCR) with Vα24Jα18 and Vβ11 chains in humans and characteristic surface markers of natural killer cells, such as CD161. iNKT cells have been implicated in the progression and resolution of several pathologies, such as infectious and allergic diseases, cancers, and autoimmune diseases, such as T1D and lupus 2.

Studies in non‐obese diabetic (NOD) mice have shown that iNKT cells play an important role in T1D development because they produce subnormal levels of IL‐4 if stimulated, and there is a decrease in their frequency 3. Adoptive transfer and activation of functional iNKT cells in NOD mice can prevent or stop disease progression. Comparable effects have been observed in NOD transgenic mice that have increased iNKT cell numbers due to iTCR overexpression 4.

iNKT cell defects that are similar to those in the NOD mouse have been suggested in humans. However, the results have been inconclusive because Wilson et al. reported a low iNKT cell frequency and an extreme Th1 bias in T1D patients 5, whereas other groups showed an increase 6 or no change in this cell population 7. Nonetheless, these studies predicted a role for iNKT cells in T1D development. However, no studies have explored the activation state of this cell population in the T1D context. Class I MHC‐restricted T cell‐associated molecule (CRTAM; also allocated as CD355 8) is a transmembrane protein that is expressed on the cell surface of mouse and human activated iNKT, CD8^+^, and CD4^+^ T cells. CRTAM expression identifies activated cellular subpopulations with pro‐inflammatory profiles, and it has been correlated with cellular processes, such as adhesion, cytotoxicity, and Th1 and Th17 cytokine production 9, 10, 11, 12. Despite its likely participation in the inflammatory immune response, its role in in vivo pathologic scenarios has been poorly explored.

Recently, Fornari et al. reported differential expression of the *crtam* gene on CD3^+^ T cells from NOD mice during T1D development 13; however, protein expression by iNKT lymphocytes has not been demonstrated in this pathology.

In this study, we compared iNKT cell frequency and CRTAM expression, as an activation marker, in a Mexican population of children with T1D and their first‐degree relatives with healthy families.

Our results showed a numerical deficiency in iNKT lymphocytes in a Mexican cohort of children who were newly diagnosed with T1D and their first‐degree relatives compared with healthy families. This frequency impairment displayed a clear familial tendency. Additionally, we provide the first evidence of activated iNKT cells downregulating iTCR and expressing CRTAM in the peripheral blood.

## Materials and Methods

### Subjects

This study involved 391 subjects, including 69 patients with recent‐onset (<3 months) T1D diagnosed according to the American Diabetes Association criteria 14 at the Pediatrics Hospital of the “Centro Medico Nacional Siglo XXI. IMSS,” 76 non‐diabetic siblings, and 116 parents. The control group included 53 healthy control subjects of similar ages, including 20 siblings and 57 parents. The detailed characteristics are included in Table 1. All subjects or their legal guardians signed informed consent forms prior to blood sample collection. Approval from the Ethics Committee of Centro Médico Nacional “Siglo XXI,” Instituto Mexicano del Seguro Social, was obtained for this research study.

**Table 1:** Study group characteristics

					AutoAb (%positive) (U/ml mean ± SE)^2^		
Groups	Total subject number	Mean age (years) ± SE	FPG (mg/dl) ± SE^1^	HbA1c (%) ± SE (mmol/mol)	GAD65^a^	IA2^b^	Insulin^c^
Newly diagnosed type 1 diabetics	69	9.6 ± 0.5	115.4 ± 7.6	8.4 ± 0.3 (68 ± 3.3)	37.7 (28.4 ± 8.5)	40.6 (126.5 ± 28.5)	30.4 (7.1 ± 1.9)
Siblings	76	13.2 ± 0.7	85.7 ± 0.8	5.3 ± 0.7 (34 ± 7.7)	32.9 (7.1 ± 1.9)	7.9 (8.45 ± 5.6)	10.5 (1.2 ± 0.5)
Parents	116	38.4 ± 0.8	109 ± 5.6	5.9 ± 0.2 (41 ± 2.2)	10.3 (2.5 ± 0.7)	6 (5.4 ± 4)	9.5 (1.2 ± 0.4)
Normal controls	53	9.8 ± 0.6	85.7 ± 1.3	5.4 ± 0.05 (36 ± 0.5)	3.7 (1.7 ± 1)	1.8 (0.4 ± 0.2)	15.1 (4.3 ± 2)
Sibling controls	20	13.1 ± 1.0	86.9 ± 2.2	5.2 ± 0.1 (33 ± 1.1)	15 (3.3 ± 2.1)	0 (0.2 ± 0.2)	10 (3 ± 1.5)
Parent controls	57	38.2 ± 1.0	97.0 ± 4.2	6.5 ± 0.7 (48 ± 7.7)	22.8 (5.9 ± 1.7)	0 (0.2 ± 0.1)	19.3 (2.4 ± 0.7)

1 FPG, Fasting plasma glucose = 126 mg/dl was considered diagnostic of diabetes.

2 Auto‐antibodies were determined by ELISA. Positive values: a = 5.0 UI/mL; b = 7.5 UI/mL; c > 1.05 U/ml.

As part of the diagnostic assessments, autoantibodies and HbA1c were determined at the Research Unit on Clinical Epidemiology (UMAE), Specialties Hospital, Centro Médico Nacional “Siglo XXI,” Mexican Social Security Institute. Auto‐antibodies were determined with commercial ELISA kits following the manufacturer's protocols. These kits included anti‐GAD (catalog number GWB‐521227) and anti‐IA2 kits (catalog number GWB‐521228) from Genway Biotech (San Diego, CA) and an anti‐insulin kit (catalog number 21‐IAAHU‐E01) from Alpco diagnostics (Salem, NH). The HbA1c percentage was determined from whole blood using ion exchange‐HPLC.

### PBMC isolation and cell staining

Heparinized peripheral blood samples were collected from the subjects, and mononuclear cells (PBMCs) were obtained by Ficoll gradient centrifugation following the manufacturer's protocol (GE Healthcare Bio‐Sciences AB, Uppsala, Sweden). PBMCs were incubated for 15 min with human γ‐globulins at 4°C for FcR‐blocking. Then, the cells were washed and immediately labeled with specific antibodies or isotype controls for 30 min at 4°C. Specifically, antibodies against the following molecules were used: Vα24‐PE and Vβ11‐FITC (Beckman Coulter, Brea, CA), CD3‐PerCPCy5.5, CD4‐PE, (BioLegend, San Diego, CA), CD8‐PB (BD Pharmingen, Franklin Lakes, NJ), CRTAM‐APC (R&D, Minneapolis, MN), and CD69‐PETR (Beckman Coulter). The following isotype controls were used: IgG1‐PE, IgG2a‐FITC, IgG1‐PerCPCy5.5 (all from BioLegend), IgG1‐PB, (BD Pharmingen), IgG2b‐APC (R&D), and IgG1‐PETR (Beckman Coulter). After incubation, the cells were washed with PBS/2% FBS and were fixed with 4% paraformaldehyde in PBS.

### Flow cytometry

Stained cells were analyzed with a CyAn ADP flow cytometer (Beckman Coulter), and approximately 1 × 10^6^ events were acquired for each sample. The data were analyzed with FlowJo (v.7.6.5, Tree Star, Inc., Ashland, OR). Lymphocytes were gated according to their light‐scattering properties. The iNKT cell percentage was obtained by gating CD3^+^Vα24^+^Vβ11^+^ triple expression. Absolute numbers of iNKT cells/ml were calculated from total blood counts. The gating strategy is shown in Suppl. Fig. S1. Vα24 and Vβ11 chain expression levels were calculated according to the mean fluorescence intensity (MFI) of each iNKT population. CRTAM and CD69 expression levels were considered as the MFI from the whole iNKT subset (iNKT^hi^ and iNKT^lo^). Differences between iNKT subpopulations were calculated as the MFI‐fold increase from the respective isotype control.

### Statistical analysis

Statistics were determined by Mann–Whitney U‐tests using GraphPad Prism Software v5 (GraphPad Prism Software Inc., La Jolla, CA). The definition of statistical significance was set at *p* < 0.05.

## Results

### Reduced iNKT cell numbers in T1D families

**Figure 1:**
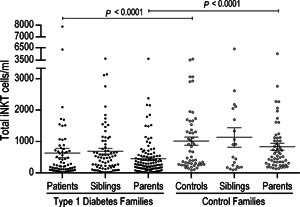
Total iNKT cell deficiency in families with type 1 diabetes. Absolute total iNKT cell numbers in type 1 diabetes families (black circles) and control families (white circles). Only significant differences between comparable groups are shown. The Mann–Whitney U‐test was utilized. The mean and SE values are indicated with a horizontal line.

We evaluated the iNKT cell frequency in T1D patients and their first‐degree relatives, and we compared these frequencies with those in healthy control families. The iNKT cell frequency (CD3^+^Vα24^+^Vβ11^+^) was analyzed as described in the “Materials and Methods” section. We did not find any differences in iNKT cell percentages (Table 2 and Supplemental Fig. S2). However, significantly lower absolute iNKT cell numbers (*p* < 0.01) were observed in the T1D patients (666.5/ml ± 167.9) compared with the healthy controls (827.5/ml ± 101). Similar differences were observed in the parents of the patients (454.7/ml ± 55.9) compared with the parents of the control group subjects (811.9/ml ± 111.5; *p* < 0.001) (Fig. 1). Although the results from the sibling groups were not significant (*p* = 0.24), a similar tendency was evident (siblings of patients 631.3/ml ± 80.6 vs. siblings of controls 1071/ml ± 324.1). These results reveal the impact of genetic background on iNKT cell numbers and suggest a probable role for these cells in T1D. It is worth to mention that the presence of auto‐antibodies did not show any correlation with iNKT cells numbers.

**Table 2:** iNKT^hi^ and iNKT^lo^ cell percentages and absolute numbers

Groups	Newly diagnosed type 1 diabetics	Siblings	Parents	Normal controls	Sibling controls	Parents controls	
Total subject number	69	76	116	53	20	57	
Subjects with iNKT^hi^	69/69	76/76	116/116	53/53	20/20	57/57	
% iNKT^hi^ cells ± SE	0.152 ± 0.02	0.219 ± 0.02	0.156 ± 0.01	0.169 ± 0.02	0.219 ± 0.07	0.154 ± 0.02	
# iNKT^hi^ cells ± SE	385.9 ± 60.6	563.1 ± 68.0	328.8 ± 33.0	827.5 ± 101.0	1071 ± 324.1	811.9 ± 111.5	
Subjects with iNKT^lo^ (%)	16/69 (23.2)	22/76 (28.9)	25/116 (21.5)	0/53	0/20	0/57	
% iNKT^lo^ cells ± SE	0.280 ± 0.10	0.147 ± 0.06	0.264 ± 0.08	–	–	–	
# iNKT^lo^ cells ± SE	1232 ± 525.1	379.2 ± 146.9	634.9 ± 196.2	–	–	–	
Subjects with NKT^lo^ and CRTAM expression (%)	12/16 (75)	19/22 (86.3)	10/15 (66.6)	–	–	–	

### Patients with T1D have two iNKT lymphocyte subpopulations

**Figure 2:**
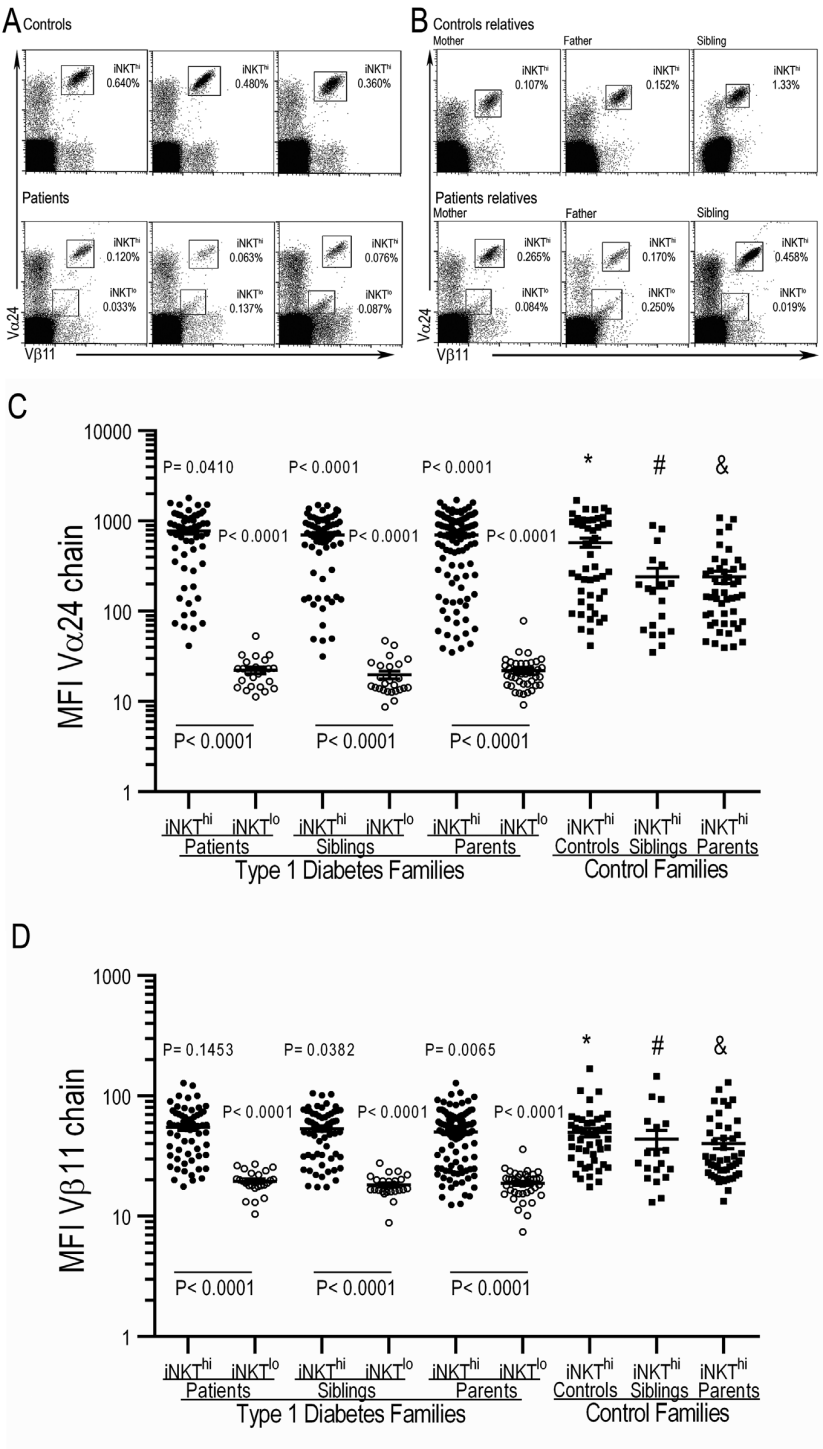
iNKT cells have low iTCR expression in type 1 diabetes patients and their first‐degree relatives. Representative dot plots from three different healthy controls and T1D patients (A) or their first‐degree relatives (B) show the iTCR expression (Va24^+^Vβ11^+^) on CD3^+^ cells. (C) Mean fluorescence intensity of the V α24 and V β11 (D) chains of the iTCR from iNKT cell populations from T1D and control families. The Mann–Whitney U‐test was utilized. The mean and SE values are indicated with a horizontal line. *Control versus patients; ^#^control siblings versus patient siblings; & parents of controls versus parents of patients.

We identified two iNKT cell subpopulations according to iTCR expression. One expressed high Vα24/Vβ11 levels (iTCR) (iNKT^hi^), and this subpopulation was present in all of the analyzed samples. The other population expressed low iTCR (iNKT^lo^) levels and was found in 16 of 69 patients (23.2%) (Fig. 2A), 22 of 76 siblings (28.9%), and 25 of 116 parents (21.5%) (Fig. 2B and Table 2). iTCR expression according to Vα24 and Vβ11 chain MFIs was significantly different between the iNKT^hi^ and iNKT^lo^ populations. Figure 2C displays the higher MFI staining for Vα24 in iNKT^hi^ compared with iNKT^lo^ subsets from patients (777.2 ± 59.3 vs. 22.3 ± 1.8), siblings (696.7 ± 52.3 vs. 19.8 ± 1.9), and parents (695.5 ± 44 vs. 22 ± 1.8) (*p* < 0.0001). Additionally, there were significant differences (*p* < 0.0001) between the iNKT^hi^ subset from control families when compared with the iNKT^lo^ cells present in T1D families (578.8 ± 69.8 in controls vs. 22.3 ± 1.8 in patients; 239.7 ± 59.5 in siblings of patients vs. 19.8 ± 1.9 in siblings of controls; and 240.1 ± 36.4 in parents of patients vs. 22 ± 1.8 in parents of controls). Similar results were observed for Vβ11 chain expression levels in iNKT^hi^ compared with iNKT^lo^ subpopulations (54.9 ± 3.3 vs. 19.5 ± 0.9 in patients, 53.4 ± 2.8 vs. 18.3 ± 0.7 in siblings, 50.2 ± 2.4 vs. 18.8 ± 0.8 in parents, respectively) (*p* < 0.0001). When comparing Vβ11 chain expression levels in iNKT^hi^ cells from control families with iNKT^lo^ cells from patients and their first‐degree relatives, the differences were as follows: 49.7 ± 4 versus 19.5 ± 0.9 between controls and patients, respectively; 43.9 ± 8 versus 18.3 ± 0.7 between siblings of controls and siblings of patients, respectively; and 40.2 ± 4 versus 18.8 ± 0.8 between parents of controls and parents of patients, respectively (*p* < 0.0001) (Fig. 2D). We also detected mild differences when comparing the iTCR chains from iNKT^hi^ populations. Specifically, we observed higher Vα24 chain expression levels and less evident Vβ11 expression in patients versus controls (777.2 ± 59.3 vs. 578.8 ± 69.8 [*p* = 0.041] and 54.9 ± 3.3 vs. 49.7 ± 4 for each respective iTCR chain), patient siblings versus control siblings (696.7 ± 52.3 vs. 239.7 ± 59.5 [*p* < 0.0001]and 53.4 ± 2.8 vs. 43.9 ± 8 [*p* = 0.0382] for each respective iTCR chain), and in parents of patients versus parents of controls (695.5 ± 44 vs. 240.1 ± 36.4 [*p* < 0.0001] and 50.2 ± 2.4 vs. 40.2 ± 4 [*p* = 0.065] for each respective iTCR chain). The frequency of the iNKT^lo^ population in healthy controls, their first‐degree relatives, and the rest of the T1D families was too low to be confidently distinguished from background staining.

### iNKT^lo^ lymphocytes are an activated subpopulation

**Figure 3:**
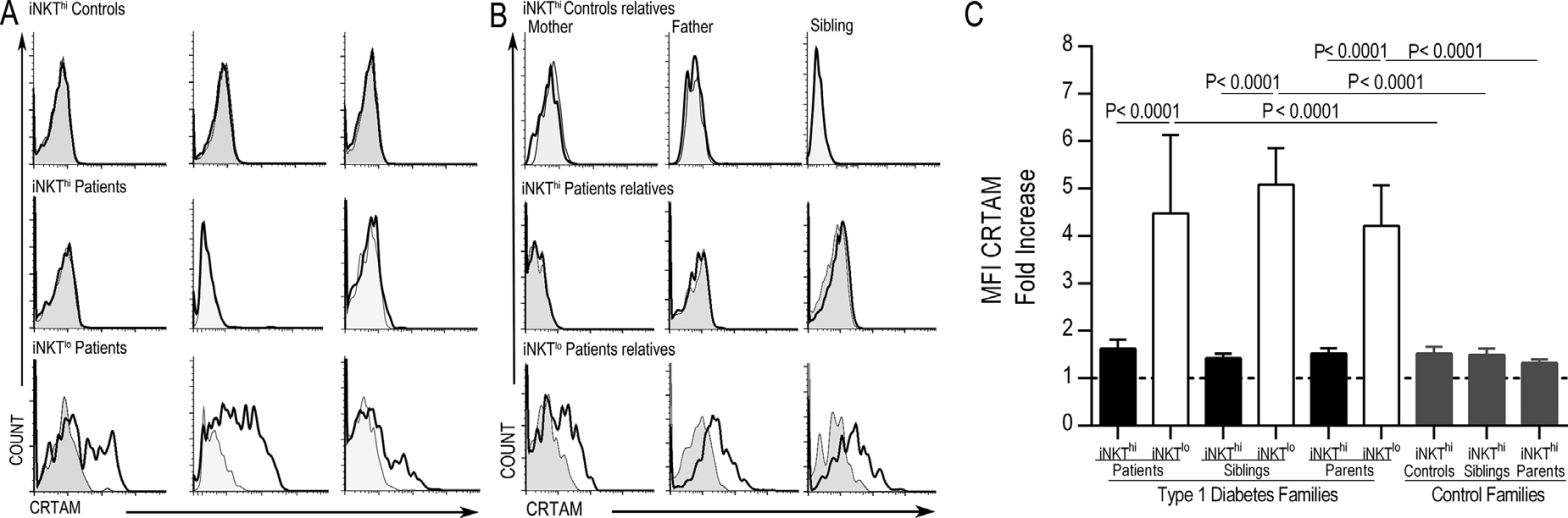
iNKT^lo^ cells express CRTAM. CRTAM expression in peripheral iNKThi and iNKTlo cells from the same three T1D patients and controls (A) or their first‐degree relatives (B) are shown. The representative histograms correspond to the same subjects as in Figure 2A and B and the gray histograms show the staining for the isotype control monoclonal antibodies. (C) CRTAM expression on iNKT^hi^ and iNKT^lo^ populations from the study groups is displayed as a fold increase. The Mann–Whitney U‐test was utilized. The mean and SE values are indicated with a horizontal line.

**Figure 4:**
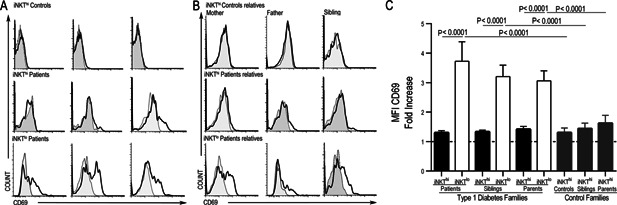
iNKT^lo^ cells express CD69. CD69 expression in peripheral iNKThi and iNKTlo cells from three patients and controls (A) or their first‐degree relatives (B) is shown. Representative histograms correspond to the same subjects as in Figure 2A and B. The gray histograms show the staining for isotype control monoclonal antibodies. (C) CD69 expression on iNKThi and iNKTlo populations from the study groups is displayed as a fold increase. The Mann–Whitney U‐test was utilized. The mean and SE values are indicated with a horizontal line.

Because the iNKT^lo^ lymphocytes resemble an activated population due to their low iTCR expression, we inferred that this population should express activation markers. CRTAM is upregulated on lymphocytes only after an activation stimulus, which is in contrast with CD69 that has low constitutive expression on human iNKT cells 15, 16. Therefore, we analyzed CRTAM and CD69 expression levels on both iNKT^hi^ and iNKT^lo^ subsets. The results showed that iNKT^lo^ cells expressed CRTAM in 75.5% of patients who had this population; however, there was no expression of this molecule on iNKT^hi^ cells (Fig. 3A and Table 2). The same expression pattern was observed in the first‐degree relatives of the T1D patients who presented the iNKT^lo^ population (Fig. 3B). After comparing the MFI fold increases amongst the iNKT populations, we observed an increase from four‐ to fivefold in MFI CRTAM expression in the iNKT^lo^ subset compared with the iNKT^hi^ subset (*p* < 0.0001) (Fig. 3C). We also detected an increase from three‐ to fourfold in CD69 MFI in the iNKT^lo^ population compared with the iNKT^hi^ population (*p* < 0.0001) (Fig. 4C). These results confirm increased activation in both patients and their first‐degree relatives that present with the iNKT^lo^ subpopulation (Fig. 4A and B). We did not observe CRTAM or CD69 expression on CD4^+^ or CD8^+^ T cells (data not shown). Although only a limited number of surface markers could be examined due to the scarcity of these cells, these results strongly suggest that iNKT^lo^ cells may be a recently activated population and thus have downregulated iTCR expression.

## Discussion

Multiple reports have attempted to determine the relevance of iNKT cell frequency in the pathogenesis of T1D in humans. However, the results have been inconclusive due to controversial findings across different populations. In addition to the genetic variability amongst the populations, almost every study has reported the percentages of iNKT cells rather than absolute numbers. Additionally, they have focused their attention on comparing iNKT cell frequencies between T1D patients and risk groups, different disease stages, and other autoimmune or endocrine pathologies, and not with healthy subjects 6, 7, 17, 18, 19, 20. Kurkreja et al. reported a lower iNKT cell frequency in T1D patients and at‐risk non‐diabetic relatives compared with normal subjects 19, and Wilson et al. compared twins and triplets and obtained similar findings 5. More recently, Montoya et al. 15 compared the iNKT cell frequency among patients and their first‐degree relatives with no observed differences. This study shows for the first time a familial decrease in iNKT cell numbers in T1D patients and their first‐degree relatives compared with healthy controls and their respective families. Regarding percentages, our results are similar to those reported by Lee et al., who showed a broad range of iNKT cell frequencies across groups and no differences amongst them 7. In a mouse model, Esteban et al. identified the two main loci (*Nkt1* and *Nkt2*) that control thymic NKT cell numbers, and they mapped them to the distal part of the *Idd13* region of chromosome 2 and the *Bana3* region of chromosome 1, respectively. Both loci have been associated with T1D and lupus development 21. Recently, it was demonstrated that the *Nkt1* gene controlled NKT numbers through the differential expression of *Slamf1* 22.

An unexpected finding during our iNKT lymphocyte frequency analysis was the presence of a population with a low iTCR density on its surface. This population resembled a population that was reported by Gadola et al. 23 after expansion of Vα24^−^/CD1d‐αGC‐tetramer^+^ T cells from PBMCs from healthy donors. Additionally, Diana et al. 24 and Constantinides et al. 25 reported a population with the same characteristics as that in our study. Lucas et al. 16 reported lower iNKT cell levels during HCV infection. Dot plots from HCV‐seropositive PCR‐positive patients showed a population with low levels of iTCR that was not present in PCR‐negative patients. However, in these papers, there was no discussion regarding the population with low iTCR levels. We strongly believe that the iNKT^lo^ population may actually be a recently activated population, as it expressed both CRTAM and CD69. The NKT^lo^ population brings to mind those reported in mice by Wilson et al. and Harada et al., which downregulated iTCR expression through endocytosis after activation and before expansion 20, 26. This type of regulation has been reported for conventional T cells 27, 28. Additionally, as CRTAM is expressed only on activated lymphocytes, the ex vivo CRTAM and CD69 expression levels on 75.5% of the iNKT^lo^ subpopulation support our hypothesis that it could be a recently activated cell population (most likely activated by autoantigens). Additionally, the fact that we observed higher iTCR levels (Vα24/Vβ11) amongst the iNKT^hi^ cells from patient families compared with controls families could be explained by the TCR replenishment and upregulation reported by other groups 29, 30, 31. Schrum et al. demonstrated that T cells commit to upregulating surface TCR expression during CD4^+^ T cell activation despite its early downregulation following antigen exposure 29, 31. They described that the new surface TCR expression level was observable several days into the response, and it increased in proportion to the antigen dose, stimulus duration, and degree of costimulation. This recovery of surface TCR expression was up to twofold higher than the original expression level 30.

It is noteworthy to mention that the age of the patients, controls, and their respective siblings limited the amount of blood samples that were obtained. Therefore, it was not possible to do further analyses, such as cell sorting or functional assays in order to deeply characterize this population. In addition, the low frequency of the cell subset limited such studies.

Recently, using a T1D mouse model induced by CD8‐OTI cells, it was shown that the interaction of CRTAM with its ligand, nectin‐like‐2 (Necl‐2), was necessary for CD8^+^ auto‐reactive lymphocyte retention in pancreatic lymph nodes and their further activation, proliferation, and differentiation into an optimal effector phenotype 12. Therefore, it is likely that the CRTAM‐Necl‐2 interaction plays a role in iNKT cell function during T1D development. However, further studies must be conducted to support this hypothesis in patients.

In conclusion, our results demonstrate that iNKT cell frequency is a family trait most likely determined by genetic factors such as those in mice. This finding suggests that this feature could be another susceptibility factor instead of a direct cause of disease. Nevertheless, a deeper genetic and prospective study is needed to establish a stronger relationship and a possible mechanism. The finding that iNKT^lo^ lymphocytes were present in a percentage of siblings and parents suggests an activation state. This factor might also contribute to T1D development susceptibility. Nevertheless, prospective studies are necessary to establish a better association and to determine the role of this lymphocyte population in the context of pathophysiology.

## Conflict of Interest

None declared.

## Supporting information

Additional supporting information may be found in the online version of this article at the publisher's web‐site.

**Figure S1:** Gating strategy for the flow cytometry analysis. PBMCs were isolated from the analyzed subjects. A–D: Gating strategy for the iNKT populations. Doublets were excluded (A), the lymphocyte population was gated based on the forward and side scatter (B), the CD3‐expressing population was selected (C), and the iNKT cell populations were gated based on their iTCR expression (Va24^+^Vβ11^+^) (D). The total iNKT cells/ml were calculated based on the percentage of iNKT^hi^ cells plus the iNKT^lo^ cells when they were present.

**Figure S2:** The percentage of total iNKT cells in type 1 diabetes (black circles) and control families (white circles). Only significant differences between comparable groups are shown. The Mann–Whitney U‐test was utilized. The mean and SE values are indicated with a horizontal line.

**Figure S3:** Isotype controls for iNKT cell flow cytometry analysis. To confirm the specificity of the antibodies used for flow cytometry, PBMCs from the analyzed subjects were stained with antibodies against iTCR (Va24^+^Vβ11^+^) (Fig. 2A) or the respective isotype controls. Representative dot plots from the same subjects as in Figure 2A are shown.
