# Supplementary figures and images for: Reduced iNKT cells numbers in type 1 diabetes patients and their first‐degree relatives
Source: Immun Inflamm Dis. 2015 Aug 18;3(4):411–9. doi: 10.1002/iid3.79 (PMC4693717; doi:10.1002/iid3.79)

A

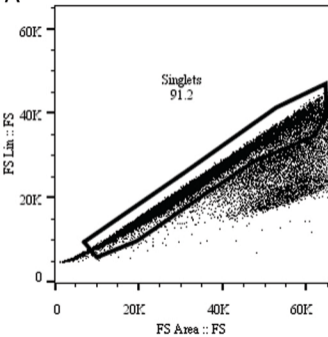

B

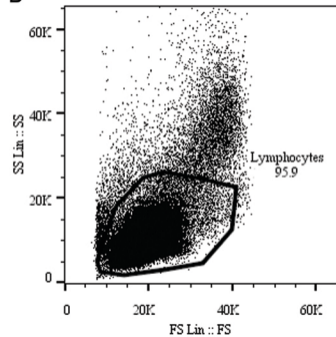

C

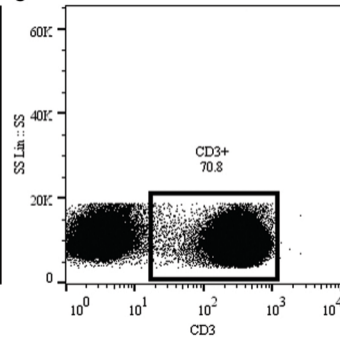

D

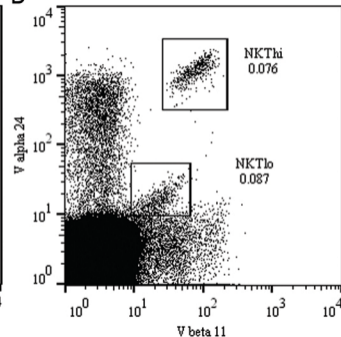

Supplement: Supplementary file 1 — Figure S1: Gating strategy for the flow cytometry analysis. PBMCs were isolated from the analyzed subjects. A–D: Gating strategy for the iNKT populations. Doublets were excluded (A), the lymphocyte population was gated based on the forward and side scatter (B), the CD3‐expressing population was selected (C), and the iNKT cell populations were gated based on their iTCR expression (Va24+Vβ11+) (D). The total iNKT cells/ml were calculated based on the percentage of iNKThi cells plus the iNKTlo cells when they were present. [file IID3-3-411-s001.pdf]

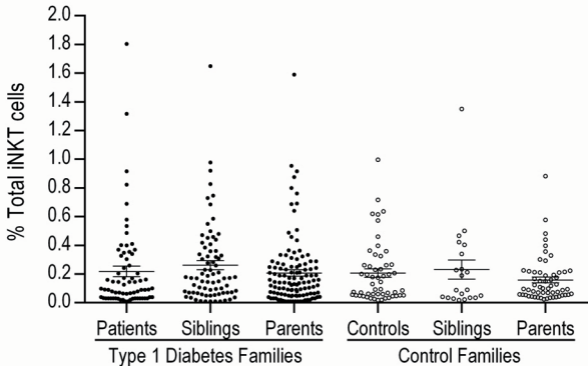

Supplement: Supplementary file 2 — Figure S2: The percentage of total iNKT cells in type 1 diabetes (black circles) and control families (white circles). Only significant differences between comparable groups are shown. The Mann–Whitney U‐test was utilized. The mean and SE values are indicated with a horizontal line. [file IID3-3-411-s002.pdf]

Controls

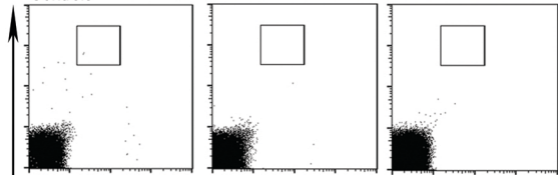

Patients

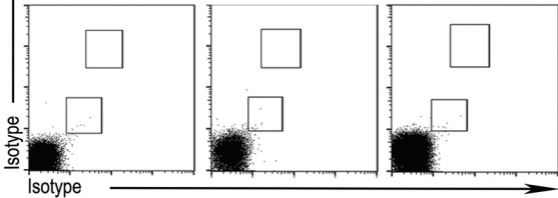

Supplement: Supplementary file 3 — Figure S3: Isotype controls for iNKT cell flow cytometry analysis. To confirm the specificity of the antibodies used for flow cytometry, PBMCs from the analyzed subjects were stained with antibodies against iTCR (Va24+Vβ11+) (Fig. 2A) or the respective isotype controls. Representative dot plots from the same subjects as in Figure 2A are shown. [file IID3-3-411-s003.pdf]
